# Inter-observer agreement according to three methods of evaluating mammographic density and parenchymal pattern in a case control study: impact on relative risk of breast cancer

**DOI:** 10.1186/s12885-015-1256-3

**Published:** 2015-04-12

**Authors:** Rikke Rass Winkel, My von Euler-Chelpin, Mads Nielsen, Pengfei Diao, Michael Bachmann Nielsen, Wei Yao Uldall, Ilse Vejborg

**Affiliations:** 1 Department of Radiology, University Hospital Copenhagen, Rigshospitalet, Blegdamsvej 9, DK-2100 Copenhagen Ø, Denmark; 2 Department of Public Health, University of Copenhagen, Øster Farimagsgade 5, DK-1014 Copenhagen K, Denmark; 3 Department of Computer Sciences, University of Copenhagen, Universitetsparken 5, DK-2100 Copenhagen Ø, Denmark; 4 Biomediq, Fruebjergvej 3, DK-2100 Copenhagen Ø, Denmark

**Keywords:** Breast cancer, Mammographic breast density, Mammographic parenchymal patterns, BI-RADS, Tabár, Interactive threshold technique, Case control study, Reproducibility, Breast cancer risk

## Abstract

**Background:**

Mammographic breast density and parenchymal patterns are well-established risk factors for breast cancer. We aimed to report inter-observer agreement on three different subjective ways of assessing mammographic density and parenchymal pattern, and secondarily to examine what potential impact reproducibility has on relative risk estimates of breast cancer.

**Methods:**

This retrospective case–control study included 122 cases and 262 age- and time matched controls (765 breasts) based on a 2007 screening cohort of 14,736 women with negative screening mammograms from Bispebjerg Hospital, Copenhagen. Digitised randomized film-based mammograms were classified independently by two readers according to two radiological visual classifications (*BI-RADS* and *Tabár*) and a *computerized interactive threshold technique* measuring area-based percent mammographic density (denoted PMD). Kappa statistics, Intraclass Correlation Coefficient (ICC) (equivalent to weighted kappa), Pearson’s linear correlation coefficient and limits-of-agreement analysis were used to evaluate inter-observer agreement. High/low-risk agreement was also determined by defining the following categories as high-risk: BI-RADS’s D3 and D4, Tabár’s PIV and PV and the upper two quartiles (within density range) of PMD. The relative risk of breast cancer was estimated using logistic regression to calculate odds ratios (ORs) adjusted for age, which were compared between the two readers.

**Results:**

Substantial inter-observer agreement was seen for BI-RADS and Tabár (κ=0.68 and 0.64) and agreement was almost perfect when ICC was calculated for the ordinal BI-RADS scale (ICC=0.88) and the continuous PMD measure (ICC=0.93). The two readers judged 5% (PMD), 10% (Tabár) and 13% (BI-RADS) of the women to different high/low-risk categories, respectively. Inter-reader variability showed different impact on the relative risk of breast cancer estimated by the two readers on a multiple-category scale, however, not on a high/low-risk scale. Tabár’s pattern IV demonstrated the highest ORs of all density patterns investigated.

**Conclusions:**

Our study shows the Tabár classification has comparable inter-observer reproducibility with well tested density methods, and confirms the association between Tabár’s PIV and breast cancer. In spite of comparable high inter-observer agreement for all three methods, impact on ORs for breast cancer seems to differ according to the density scale used. Automated computerized techniques are needed to fully overcome the impact of subjectivity.

## Background

Breast cancer is the most common cancer among women worldwide and a leading cause of cancer death [1].

Breast density has been demonstrated to be one of the strongest risk factors for breast cancer [2,3]. A meta-analysis by V. A. McCormack et al. showed that women with increased mammographic density (>75%) have a four to six-fold increased risk of breast cancer compared with women with low breast density (<5%) [4]. Besides being an independent marker of breast cancer risk, density affects mammographic sensitivity by the “masking effect” and is associated with increased risk of interval cancers [2,5,6]. Moreover, breast density is known to be affected by hormonal status and has the potential of being modulated [7-10]. Integration into existing risk models like the Gail model [11] has been discussed [3,12,13] as well as density patterns forming the basis of individualized screening [2,6,14-16]. Thus, mammographic breast density is considered an important variable in cancer diagnostics, risk estimation, and possible risk modelling.

One of the key questions has been how to measure mammographic density most accurately, reliably, and simply. Basically, there are two different approaches: 1) the qualitative morphological approach based on structural information and 2) the quantitative approach which considers the amount of fibroglandular (radio dense) tissue in the breast, often expressed as a *percentage area* of dense tissue [17]. In 1976 Wolfe proposed a classification based on four different parenchymal patterns [18] which was modified into five categories by László Tabár in 1997 [19,20]. Today, the BI-RADS density classification (with a quantitative percentage graduation in the 4^th^ edition from 2003) is globally the most commonly used density classification in clinical settings, and is covered by legislation in several U.S. states [21,22]. However, inter- and intra-observer reproducibility are of great concern regarding the visual classifications [23-28]. Hence, partially and fully-automated computerized techniques are an area of active research. Several computer-aided techniques exist where the interactive area-based commercialized Cumulus software is most commonly used [29]. However, subjectivity is still not completely eliminated by the partially-automated techniques. Thus, research has in recent years focused more intensively on a fully automated objective assessment of breast density, including volumetric measures, in line with breast imaging moving from analogue to digital mammography [30-33]. In addition, density assessment carried out using other imaging modalities as digital breast tomosynthesis (DBT) or MRI are also being investigated [34,35].

As part of an ongoing research project validating a new automated computerised density score and a new automated texture score for digitized film-based mammograms, we wanted to validate the corresponding subjective visual methods of categorising density and paranchymal pattern in terms of the BI-RADS density classification, the Tabár classification on parenchymal patterns and a new partially-computerized interactive threshold technique (Cumulus-like). The reproducibility of BI-RADS has in previous papers demonstrated moderate to substantial agreement [23-25,28]. However, the reproducibility of the Tabár classification is less well described and *inter*-observer differences have to our knowledge not been reported previously. The objectives of this study were to report inter-observer agreement regarding three subjective ways of assessing density and parenchymal pattern of the female breast and to investigate where disagreement primarily occurs. Secondarily, we wanted to examine what potential impact reproducibility has on relative risk estimates of breast cancer in terms of odds ratios.

## Methods

### Population and mammograms

This retrospective case–control study is based on all 14,736 women with negative film-based screening mammograms attending biennial routine breast screening in 2007 at one specific hospital (Bispebjerg Hospital) in Capital Region, Denmark. The women were followed until death, emigration and/or occurrence of histologically verified breast cancer or ductal carcinoma in situ (DCIS) in the period between the screening dates until the end of the study on 31 December 2010. Information on death and emigration was retrieved from the Danish Civil Registration System (CRS) and information on breast cancer/DCIS was retrieved from the Danish Cancer Registry and the Danish Breast Cancer Cooperative Group (DBCG). Linkage between registers was based on the unique personal identification numbers allocated to all persons with a permanent address in Denmark.

A total of 132 women were diagnosed with breast cancer (invasive cancer and/or DCIS) in the study period. Each case was age-matched (by year of birth) with two controls from the screening cohort using incidence density sampling, i.e. the controls for each case were chosen from women who had not developed a breast cancer at the specific time when the case was diagnosed (264 controls). Film-based mammograms were not accessible for 12 women (10 cases and 2 controls) either because images were missing from the hospital’s film archive (nine women) or because only digital mammograms were available (three women). No women were additionally excluded leaving a total of 384 women for the final analyses.

Analogue mammograms of each breast were acquired in both the craniocaudal (CC) and the mediolateral oblique (MLO) projection in all but 4 cases. We ended up with 757 CC and 765 MLO views corresponding to 382 right and 383 left mammograms all together. The film-based mammograms were digitised using a Vidar Diagnostic PRO Advantage scanner (Vidar systems corporation, Herdon, VA, USA) providing an 8-bit (256 grey scales) output at a resolution of 75 DPI or 150 DPI. Images were displayed on a regular PC monitor. For tumour diagnostics these settings would be inadequate. They were, however, sufficient for our readings of breast density and parenchymal pattern.

The use of screening data and tumour-related information was approved by the Danish Data Inspection Agency (2013-41-1604). This is an entirely register based study and hence neither written consent nor approval from an ethics committee was required under Danish Law.

### Mammographic density measurements

The digitised mammograms were randomized according to case/control-status and reviewed independently by two medical doctors: a senior radiologist specialized in breast-imaging and mammography screening (Reader 1) and a resident in radiology (Reader 2). All images were analysed without knowledge of the original mammographic reading, the date of examination, the woman’s age or case/control status. The following three subjective density and parenchymal pattern classifications were investigated:

#### The BI-RADS density classification

Mammograms were classified after the *Breast Imaging Reporting and Data System* (BI-RADS) categorization on density (4^th^ edition, 2003) as defined by The American College of Radiology (ACR) [21]. The classification comprises four descriptive categories with corresponding quantitative percentage quartiles of the amount of fibroglandular tissue: *D1*: Fatty (<25% fibro-glandular tissue), *D2*: Scattered fibro-glandular densities (25-50%), *D3*: Heterogeneously dense (51-75%), *D4*: Extremely dense (>75%).

#### The Tabár classification on parenchymal patterns

**Figure 1:**
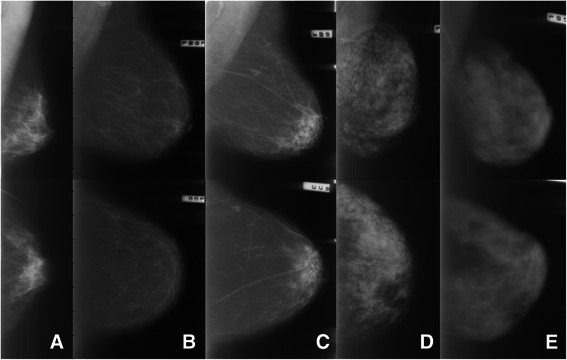
Examples of the five different parenchymal patterns (PI-PV) based on the definition by Tabár. PI-PV are shown from left to right; MLO views in the top row and CC views in the lower row. **(A)** PI: Scalloped contours with oval-shaped lucent areas and evenly scattered 1–2 mm nodular densities. **(B)** PII: Almost complete fatty replacement. **(C)** PIII: Like PII but with a retroareolar prominent duct pattern. **(D)** PIV: Dominated by extensive nodular and linear densities with nodular densities larger than normal lobules. **(E)** PV: Dominated by homogeneous, ground glass like and structure-less densities.

The Tabár classification is based on an anatomic-mammographic correlation [20]. In brief, Tabár concentrates on four basic structures: Nodular densities, linear densities, homogeneous structure-less densities, and radiolucent (dark) areas. The parenchymal pattern is categorized into the following five patterns (Figure 1) based on the relative proportion and appearance of these basic structures: *PI*: All four structures are almost equally represented with evenly scattered terminal ductal lobular units (1–2 mm nodular densities), scalloped contours and oval-shaped lucent areas. *PII*: Almost complete fatty replacement dominated by radiolucent adipose tissue and linear densities. *PIII*: Similar in composition to PII except from a retroareolar prominent duct pattern. *PIV*: Predominance of enlarged nodular densities and prominent linear densities (represent proliferating glandular structures that are considerably larger than the normal lobules and periductal fibrosis). *PV*: Homogeneous, ground glass like, structure-less fibrosis with convex contours [19,20].

#### The interactive threshold technique (percentage mammographic density, PMD)

Percentage density measurements were retrieved by a computer-aided interactive threshold technique. At first the reader distinguished the breast from the background by outlining the breast boundary and the pectoral muscle. Secondly, the reader chose the most optimal threshold separating the dense tissue from the non-dense tissue. The brightness of each pixel is represented by a grey-level (intensity) value, and pixels with intensity above or below the chosen threshold are identified accordingly as dense or non-dense tissue. PMD was computed by dividing the total number of dense pixels by the total number of pixels within the breast area, then multiplied by 100 [36].

The experienced senior radiologist had long-term experience in the use of BI-RADS but none of the other classifications had been used before by any of the readers. ACR recommendations on breast density (4^th^ edition) with the accompanying reference images as well as the classification criteria and reference images from László Tabár et al’s textbook on the Tabár patterns from 2005 were provided [20,21]. Moreover, the readers did consensus scores on a series of 66 training mammograms from 2005 regarding the Tabár classification.

In visual assessment of breast density the fibroglandular tissue should be regarded more as a volume rather than an area [25]. Thus, the CC and MLO projection were evaluated together to be able to estimate the volume of dense tissue. Readings of one breast-side of all the women were completed before scoring the opposite breasts (never evaluating a woman’s right and left breast together). Accordingly, the right and the left breasts were scored separately and can thus be considered independent measurements. Readings by the three different methodologies were completed separately at different times over a period of six months in a MatLab scoring-database. In order to further reduce artificial agreement between the methods, the readers were blinded from evaluations by the other classifications.

### Statistical analysis

An average of the MLO and CC view was used as an approximation of the most accurate measure of PMD [37]. Correlations between MLO and CC views were high (absolute agreement ICC: 0.89 and 0.93 and Pearson Correlation: 0.92 and 0.96 for each reader, respectively). Estimated CC measures were calculated from linear regression analysis for the four women where only MLO projections were available. Regarding the visual scores categorization was based on the MLO image alone for these four women as would be the case in a clinical setting.

#### Inter-observer agreement

Inter-observer consistency was investigated on both a multiple-category scale and on a high/low-risk scale. Dichotomous re-classification was done by defining the following categories as high-risk density: BI-RADS: D3 and D4, Tabár: PIV and PV and the upper two quartiles of PMD (four groups with equal percentage density ranges within density range, corresponding to the BI-RADS classification). Concordance was investigated based on all 765 independently scored right and left breast mammograms as well as on the overall scores of the 384 women (mimicking clinical praxis). In line with the BI-RADS recommendations the highest category was chosen if a woman had different density on the left and right side [38]. The Tabár patterns PIV and PV are categorized as high-risk patterns by Tabár himself but no further detailed ranking is reported [19,20,27]. One study has demonstrated increased risk of breast cancer only for pattern IV in an Asian population [39]. Based on risk evaluation from these previous studies we ranked the Tabár classification as follows: *PII, PIII, PI, PV, PIV* where the low-risk patterns PI-PIII were ranked based on increasing density. Equal to BI-RADS we also used the denser breast to assess the woman’s final score with respect to the PMD measurements.

Absolute agreement, agreement within each category and disagreement between pair wise categories were calculated. Kappa statistic was used to evaluate inter-observer agreement on BI-RADS and Tabár for multiple- and dichotomized ratings, where Cohen’s kappa indicates the proportion of agreement beyond that expected by chance. The absolute Intraclass Correlation Coefficient (ICC; two-way random, single measure), which is equivalent to the weighted kappa, was also used to measure agreement where the degree of disagreement is taken into account regarding the ordinal BI-RADS scale [40]. As suggested by Landis and Koch the strength of agreement beyond chance for different κ values is *Poor* (<0), *Slight* (0–0.20), *Fair* (0.21-0.40), *Moderate* (0.41-0.60), *Substantial* (0.61-0.80) and *Almost perfect* (0.81-1.00) [41]. Bootstrapping was used to calculate 95% confidence intervals (Cl) for kappa values using 1000 replications. Absolute ICC (two-way random, single measure), Pearson’s linear correlation coefficient (R) and limits-of-agreement analysis were calculated to analyze inter-observer reliability for the continuous PMD measures.

#### Relative risk of breast cancer

The association between mammographic density/parenchymal pattern and breast cancer risk was estimated using logistic regression to calculate odds ratios (OR) adjusted for the woman’s age at screening. Due to the retrospective design of this study, information on body mass index (BMI) and other breast cancer risk variables could not be obtained and controlled for. PMD measured by the threshold technique was divided into four equal percentage ranges—quartiles within range of the PMD measures—corresponding to the BI-RADS categorization into density quartiles. For all methods the higher density groups were compared individually with the lowest density group (baseline). Accordingly, D1 was used as reference category for BI-RADS, PII for Tabár and the lowest quartile for PMD.

Exact two-sided P-values and 95% confidence intervals (95% CI) have been listed and results were considered statistically significant with P-values ≤ 0.05.

IBM SPSS Statistics 20, Copyright © IBM Corporation 1989–2011, was used for statistical analysis.

## Results

### Characteristics of cases and controls

The women were aged between 50 and 69 years (mean age of cases 57.8 (SEM 0.49) and controls 58.1 (SEM 0.34), respectively). In total 110 women were diagnosed with invasive cancer and 12 with ductal carcinoma in situ (DCIS). Breast cancer was diagnosed < 12 months after the negative 2007-screening in 15 women, between 12–24 months in 22 women, and > 24 months in 85 women, respectively.

### Inter-observer agreement

#### The BI-RADS density classification

**Figure 2:**
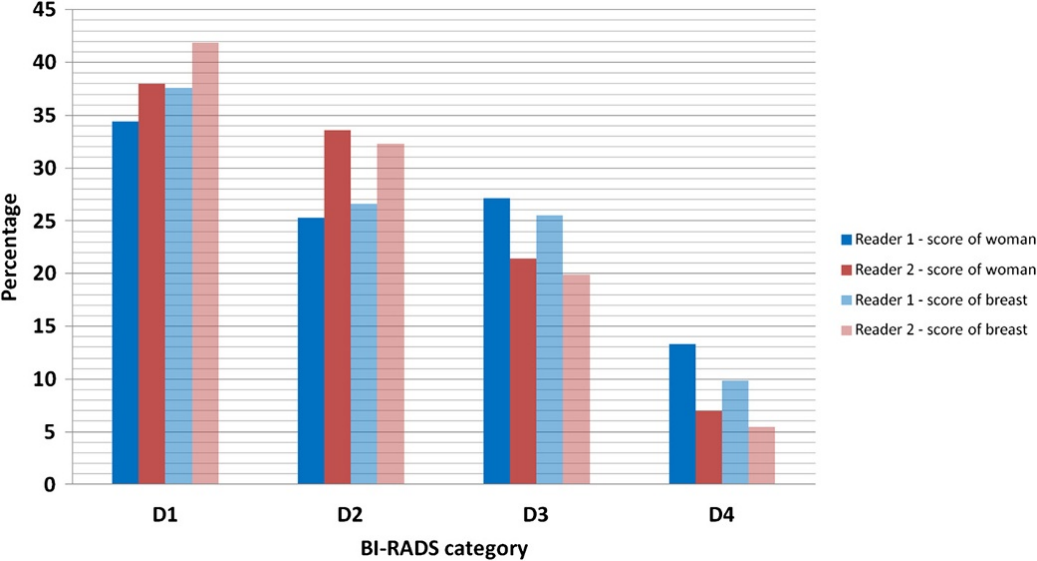
Percentage distribution of BI-RADS categories reported by Reader 1 and 2. Data are shown based on score of the women* (n = 384) and of each breast** (n = 765). *Highest category if different categories were assessed on the left and the right breast. **Left and right mammograms were scored independently and CC and MLO views evaluated together.

The percentage distribution on BI-RADS categories reported by the two readers is shown in Figure 2. Reader 1 (R1) regarded significantly more as having a high-risk density pattern (D3 and D4) compared with Reader 2 (R2) (155 (40%) versus 109 (28%) women). The proportion of women consistently classified with a high-risk pattern among the two readers was 28%.

Table 1 demonstrates the agreement between the two readers in a cross table. Consistency was highest for low risk patterns with the following agreement within each D1-D4 BI-RADS category: 94%, 72%, 62% and 69%, respectively. Two-grade disagreement was only seen in one case (D2/D4) corresponding to 0.1% (breast based). R1 judged systematically one category higher regarding 157 of the 765 disagreed breast mammograms (21%), and only 2% were judged in a lower category compared with R2.

**Table 1:** **Inter-observer agreement on the BI-RADS density classification**

	Reader 2					
Reader 1	*D1*	*D2*	*D3*	*D4*	*Total*	*High/low-risk*
***D1***	**131** **(282)**	1 (7)	0 (0)	0 (0)	132 (289)	229; 60%
***D2***	15 (40)	**81** **(160)**	1 (4)	0 (0)	97 (204)	(493; 64%)
***D3***	0 (0)	46 (80)	**58** **(113)**	0 (3)	104 (196)	155; 40%
***D4***	0 (0)	1 (1)	23 (36)	**27** **(39)**	51 (76)	(272; 36%)
***Total***	146 (322)	129 (248)	82 (153)	27 (42)	384 (765)	P<0.0001
***High/low-risk***	275; 72% (570; 75%)		109; 28% (195; 25%)			
**Agreement**	**Women (%)**		**Breasts (%)**			
*Absolute agreement:*	77.3		77.6			
*D1/D2 disagreement:*	4.2		6.1			
*D2/D3 disagreement:*	12.2		11.0			
*D3/D4 disagreement:*	6.0		5.1			
*Two-grade disagreement:*	0.3		0.1			
*High/low-risk agreement:*	87.5		88.9			

Based on 384 women (breasts are shown in brackets; n=765).

Numbers in boldface indicate agreement between the two readers.

Kappa statistics on inter-observer agreement are shown in Table 2. Agreement was substantial for side based assessment (κ = 0.68) and almost perfect when calculating the weighted kappa measured by ICC (0.88). High/low-risk categorization showed some increase in agreement (κ = 0.74). Inter-observer agreement tended to be highest for controls and for left-side mammograms (NS).

**Table 2:** **Kappa (κ)-statistics according to the BI-RADS and Tabár classification**

	Agreement absolute (%)	Total κ (95% CI)	Cases κ (95% CI)	Controls κ (95% CI)	Left κ (95% CI)	Right κ (95% CI)	Total ICC* (95% CI)
**Breasts**	**n=765**	**n=765**	**n=242**	**n=523**	**n=383**	**n=382**	**n=765**
**BI-RADS**							
***4-categories***	77.6	0.68 (0.64-0.72)	0.65 (0.57-0.73)	0.69 (0.64-0.74)	0.71 (0.66-0.77)	0.65 (0.59-0.71)	0.88 (0.81-0.92)
***Low/high-risk***	88.9	0.74 (0.68-0.79)	0.75 (0.66-0.83)	0.72 (0.65-0.78)	0.74 (0.66-0.81)	0.75 (0.67-0.82)	-
**Tabár**							
***5-categories***	74.5	0.64 (0.60-0.69)	0.56 (0.47-0.63)	0.67 (0.62-0.72)	0.70 (0.64-0.75)	0.59 (0.53-0.65)	-
***Low/high-risk***	88.2	0.70 (0.63-0.80)	0.72 (0.63-0.80)	0.67 (0.58-0.75)	0.75 (0.69-0.82)	0.65 (0.55-0.73)	-
**Women**	**n=384**	**n=384**	**n=122**	**n=262**			**n=384**
**BI-RADS**							
***4-categories***	77.3	0.68 (0.63-0.74)	0.60 (0.49-0.71)	0.72 (0.65-0.78)			0.89 (0.79-0.93)
***Low/high-risk***	87.5	0.73 (0.66-0.79)	0.69 (0.57-0.81)	0.73 (0.63-0.81)			-
**Tabár**							
***5-categories***	74.7	0.65 (0.59-0.71)	0.55 (0.44-0.67)	0.69 (0.61-0.75)			-
***Low/high-risk***	89.6	0.77 (0.70-0.84)	0.80 (0.69-0.90)	0.73 (0.63-0.83)			-

Kappa values are based on 765 breasts and 384 women, respectively.

*ICC (two-way random, single measure) corresponding to the weighted kappa value.

#### The Tabár classification

**Figure 3:**
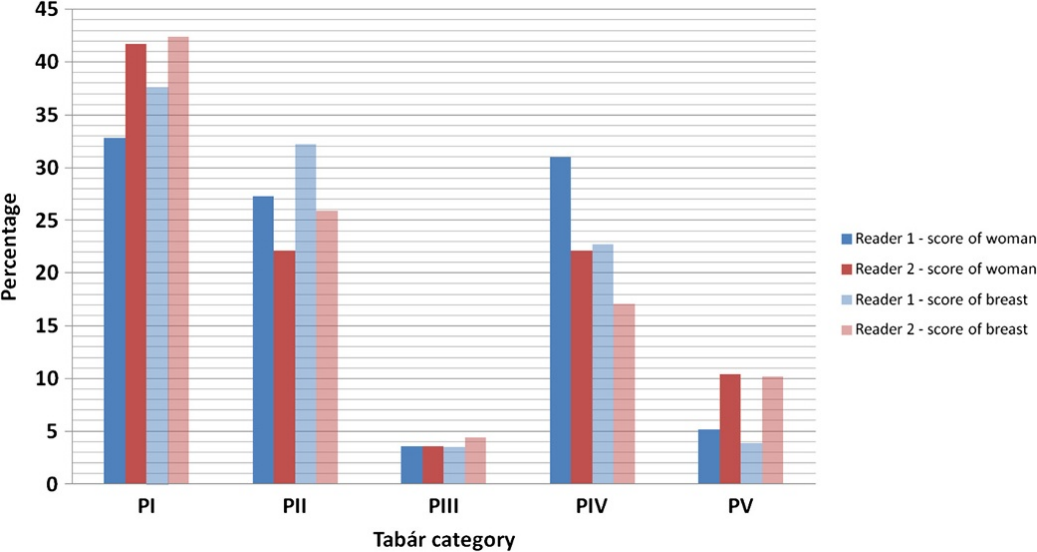
Percentage distribution of Tabár categories reported by Reader 1 and 2. Data are shown based on score of the women* (n = 384) and of each breast** (n = 765). *Highest category was selected if different categories were reported on the left and the right side (ranking: *PII, PIII, PI, PV, PIV*). **Left and right mammograms were scored independently and CC and MLO views evaluated together.

In Figure 3 the percentage distribution on Tabár patterns is shown. No statistically significant difference between readers on overall distribution was found (high-risk R1: 139 (36%) vs high-risk R2: 125 (33%) women). However, only 29% of the women would consistently be classified with a high-risk Tabár pattern by both readers.

Agreement between the two readers is shown in Table 3 including pair wise disagreement among all five categories. The concordance within each Tabár category (PI-PV) on women based evaluations was 75%, 85%, 36%, 75% and 60%, respectively. Disagreement was in most cases associated with Pattern I, where 98 breasts classified as PI by R2 were assessed as primarily PII (47) or PIV (42) by R1. Additionally, R1 classified 61 breasts as PI which were classified primarily as PV (24) or PIV (22) by R2.

**Table 3:** **Inter-observer agreement on the Tabár classification**

	Reader 2						
Reader 1	*PI*	*PII*	*PIII*	*PIV*	*PV*	*Total*	*High/low-risk*
***PI***	**107** **(227)**	2 (4)	5 (11)	7 (22)	5 (24)	126 (288)	245; 64%
***PII***	20 (47)	**81** **(191)**	4 (8)	0 (0)	0 (0)	105 (246)	(561; 73%)
***PIII***	6 (8)	2 (3)	**5** **(15)**	1 (1)	0 (0)	14 (27)	
***PIV***	26 (42)	0 (0)	0 (0)	**76** **(108)**	17 (24)	119 (174)	139; 36%
***PV***	1 (1)	0 (0)	0 (0)	1 (0)	**18** **(29)**	20 (30)	(204; 27%)
***Total***	160 (325)	85 (198)	14 (34)	85 (131)	40 (77)	384 (765)	P<0.0001
***High/low-risk***	259; 67% (557; 73%)			125; 33% (208; 27%)			
**Agreement**	**Women (%)**		**Breasts (%)**				
*Absolute agreement:*	74.7		74.5				
*PI/PII disagreement:*	5.7		6.7				
*PI/PIII disagreement:*	2.9		2.5				
*PI/PIV disagreement:*	8.6		8.4				
*PI/PV disagreement:*	1.6		3.3				
*PII/PIII disagreement:*	1.6		1.4				
*PII/PIV disagreement:*	0		0				
*PII/PV disagreement:*	0		0				
*PIII/PIV disagreement:*	0.3		0.1				
*PIII/PV disagreement :*	0		0				
*PIV/PV disagreement :*	4.7		3.1				
*High/low-risk agreement :*	89.6		88.2				

Based on 384 women (breasts are shown in brackets; n=765).

Numbers in boldface indicate agreement between the two readers.

Tabár’s 5-category scale also showed substantial agreement for breast based scoring with κ = 0.64 increasing to 0.70 using high/low-risk categorization (Table 2). Corresponding kappa values for woman based scoring were even higher, but agreement remained substantial (5-category: 0.65, 2-category: 0.77). On a multiple-category scale substantial agreement was seen among controls (0.67), while only moderate agreement was seen among cases (0.56; NS). On the contrary, the opposite tendency was seen using only two categories. Resembling assessment by BI-RADS inter-observer agreement tended to be highest on left side mammograms (left: 0.69 versus right: 0.59; NS).

#### The interactive threshold technique

**Figure 4:**
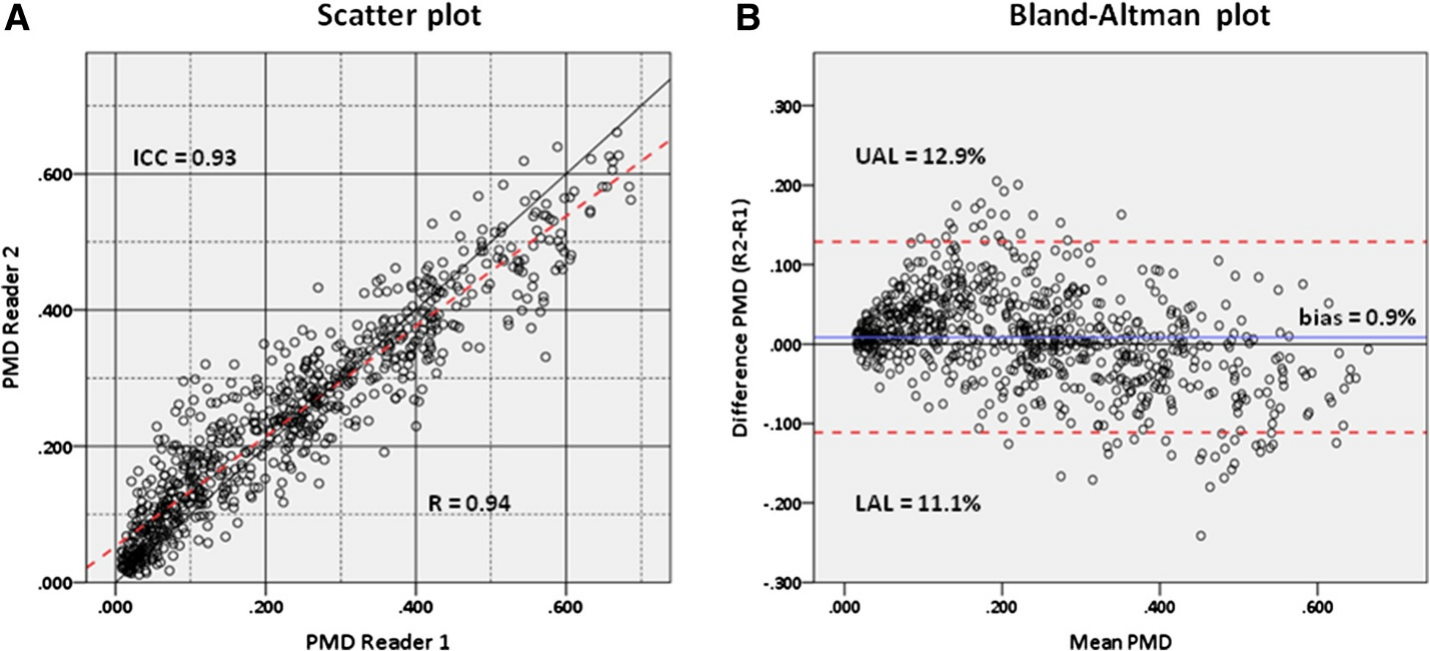
Inter-observer agreement on the interactive threshold technique. **(A)** Scatter plot illustrating the inter-observer correlation (Reader 1 *x*-axis, Reader 2 *y*-axis) of the percentage mammographic density measures (PMD) by the interactive threshold technique based on 765 breasts*. The black diagonal line indicates perfect agreement between the two readers. The red dashed line is the line of best fit. **(B)** Bland-Altman plot illustrating inter-observer agreement. Difference in PMD measures (Reader 2 minus Reader 1) is plotted against the mean PMD. The blue line shows a bias of 0.009 (≈1%) indicating only slightly higher PMD measures by R2 on average. The upper (UAL) and lower (LAL) 95% agreement limits are illustrated by the red dashed lines. *Each PMD measure is an average of the CC and MLO value. Only the MLO view was available in 8 breasts. These have been included with a corrected value after linear regression analysis.

Figure 4 shows a scatter plot of the relationship between the PMD scores by the two readers and a Bland-Altman plot illustrating the level of agreement based on 765 breasts. A high linear dependence were found with a Pearson’s correlation coefficient of 0.94 (0.93-0.95) and the readers demonstrated almost perfect agreement with an absolute ICC = 0.93 (0.92-0.94). Only a minor mean difference was seen between the readers with a negligible positive bias of 0.9% (0.4%-1.3%) for R2. Limits-of-agreement analysis with 95% limits found that the readers scored from 11.1% lower till 12.9% higher of each other. Thus, at least 95% of the PMD differences were within the range of one PMD quartile (≈16%). Both plots illustrate that R1 tended to score a little lower than R2 in fatty breasts but, on the other hand, a little higher in breasts with more glandular tissue.

Overall no statistical significant difference on distribution was found on a quartile based high/low-risk categorization (high-risk R1: 110 (29%) versus high-risk R2: 117 (30%) women), and 27% of the women were consistently classified with a high-risk pattern by the two readers.

No significant difference in inter-observer agreement was seen for cases and controls (ICC = 0.93 versus 0.92). Again consistency tended to be highest on the left side (left ICC = 0.94 versus right 0.91; NS).

### Relative risk of breast cancer

Table 4 summarizes the age-adjusted breast cancer odds ratios associated with the Tabár patterns as well as increasing mammographic density (BI-RADS and PMD) assessed by each of the two readers. A stepwise increase in relative risk with increasing density characterized by BI-RADS was seen for both readers. Likewise, a general increase in ORs with increasing density by the interactive threshold technique was seen. However, the Q4 OR of 2.17 (95% CI 0.98-4.81) was non-significant for Reader 1.

**Table 4:** **Association between breast density/parenchymal pattern and breast cancer**

	Cases (n)	Controls (n)	Cancer ratio	OR (95% Cl)*	P
**BI-RADS** **Reader 1**					
**D1**	29	103	22.0	1.00 (reference)	-
**D2**	25	72	25.8	1.25 (0.67-2.31)	0.482 (NS)
**D3**	42	62	40.4	2.47 (1.39-4.39)	0.002
**D4**	26	25	51.0	3.87 (1.91-7.85)	<0.001
***D1+D2***	*54*	*175*	*23.6*	*1.00 (reference)*	*-*
***D3+D4***	*68*	*87*	*43.9*	*2.58 (1.64-4.04)*	*<0.001*
**Reader 2**					
**D1**	32	114	21.9	1.00 (reference)	-
**D2**	39	90	30.2	1.57 (0.91-2.72)	0.106 (NS)
**D3**	38	44	46.3	3.17 (1.74-5.76)	<0.001
**D4**	13	14	48.1	3.45 (1.45-8.25)	0.005
***D1+D2***	*71*	*204*	*25.8*	*1.00 (reference)*	*-*
***D3+D4***	*51*	*58*	*46.8*	*2.56 (1.59-4.10)*	*<0.001*
**Tabár** **Reader 1**					
**PI**	34	92	27.0	1.56 (0.83-2.92)	0.168 (NS)
**PII**	20	85	19.0	1.00 (reference)	-
**PIII**	5	9	35.7	2.36 (0.71-7.81)	0.160 (NS)
**PIV**	59	60	49.6	4.14 (2.26-7.61)	<0.001
**PV**	4	16	20.0	1.04 (0.31-3.48)	0.955 (NS)
***PI+PII+PIII***	*59*	*186*	*24.1*	*1.00 (reference)*	*-*
***PIV+PV***	*63*	*76*	*45.3*	*2.61 (1.67-4.07)*	*<0.001*
**Reader 2**					
**PI**	49	111	30.6	3.31 (1.58-6.95)	0.002
**PII**	10	75	11.8	1 (reference)	-
**PIII**	6	8	42.9	5.62 (1.61-19.62)	0.007
**PIV**	43	42	50.6	7.69 (3.49-16.91)	<0.001
**PV**	14	26	35.0	4.05 (1.59-10.30)	0.003
***PI+PII+PIII***	*65*	*194*	*25.1*	*1.00 (reference)*	*-*
***PIV+PV***	*57*	*68*	*45.6*	*2.51 (1.59-3.97)*	*<0.001*
**Percentage density** Reader 1**					
**Q1**	40	122	24.7	1 (reference)	-
**Q2**	36	76	32.1	1.45 (0.85-2.49)	0.173 (NS)
**Q3**	32	44	42.1	2.24 (1.24-4.02)	0.007
**Q4**	14	20	41.2	2.17 (0.98-4.81)	0.056 (NS)
***Q1+Q2***	*76*	*198*	*27.7*	*1.00 (reference)*	*-*
***Q3+Q4***	*46*	*64*	*41.8*	*1.87 (1.17-3.00)*	*0.009*
Reader 2**					
**Q1**	28	95	22.8	1 (reference)	-
**Q2**	45	99	31.3	1.55 (0.89-2.68)	0.120 (NS)
**Q3**	33	56	37.1	2.03 (1.10-3.74)	0.023
**Q4**	16	12	57.1	4.65 (1.93-11.16)	0.001
***Q1+Q2***	*73*	*194*	*27.3*	*1.00 (reference)*	*-*
***Q3+Q4***	*49*	*68*	*41.9*	*1.92 (1.21-3.07)*	*0.006*

Relative risk estimates in terms of ORs from assessment by three subjective scoring methods by Reader 1 and 2.

*Adjusted for age.

**PMD grouped in quartiles with cut offs within density range: R1 (%): Q1) 0.99-17.89, Q2) 17.90-34.80, Q3) 34.81-51.71, Q4) 51.72-68.62; R2 (%): Q1) 1.51-17.66, Q2) 17.67-33.81, Q3) 33.82-49.96, Q4) 49.97-66.12.

According to the Tabár patterns both readers demonstrated a high OR associated with PIV of 4.14 (2.26-7.61) and 7.69 (3.49-16.91) by Reader 1 and 2, respectively. R1 found no other Tabár patterns to be significantly associated with breast cancer, whereas, R2 demonstrated increased odds ratios for all other patterns. When high-risk density patterns were combined odds ratios became more uniform among the readers but also among all three methods.

## Discussion

Even though inter-observer differences exist when assessing density or parenchymal pattern manually, the question is how much impact this has on relative risk estimates for breast cancer? Overall, this study showed a rather high (substantial to almost perfect) inter-observer agreement for all three methods investigated, which all seemed to capture the association with breast cancer assessed by both readers. However, the number of women classified with a high-risk density pattern did vary between the readers, and a different trend in disagreement for the three methods was seen leading to differences in OR-estimates by the two readers.

### BI-RADS

We found inter-observer agreement on BIRADS to be comparable with previous studies reporting k-statistics ranging from the extremes of 0.02-0.87 [23-26,42]. Observer differences rely primarily on various training as well as the reader’s experience as a breast radiologist and with the classification method, and in general moderate to substantial agreement is found (highest values for the weighted kappa/ICC). As one would expect concordance increased to some extent (NS) on a two-scale basis (from κ = 0.68-0.74). Likewise, Ciatto et al. and Bernadi et al. found substantial agreement on a two-category basis of κ = 0.71 (average of 12 readers) and κ = 0.72-0.76 (range of six readers), respectively [23,25].

The differentiation into high/low-risk categories is central as it has been suggested to form the basis of personalized screening with particular attention to the masking effect [6,23]. Mammographic sensitivity decreases in line with increasing breast density due to superposition of overlapping normal breast tissue and potential breast lesions. This masking effect on two-dimensional images leads to increased risk of interval cancers. Accordingly, women with high density may benefit from supplementary exams with e.g. digital breast tomosynthesis in which the breast is viewed in “slices” or “slabs”. Although, our results indicate a relatively high concordance, disagreement was seen to be most pronounced for the borderline D2/D3 categories and consistency was lowest within the D3 category (62%). This finding is supported by other studies on reproducibility showing that agreement is lowest in the BI-RADS density 3 category [24,42] and most evident for D2-D3 categorization [23,25]. If the women of this study were to be offered differentiated follow-up based on high-low risk from density estimates on their negative screening mammogram, 13% of the women would have been allocated differently by the two readers. In our case Reader 1 systematically judged one category higher than Reader 2 when disagreeing. An extended set of reference images or a proficiency test (as suggested by Ciatto et al. [25]) or joint training could have increased uniformity in how to perceive density, and may have improved consistency.

### Tabár

This is to our knowledge the first study to report *inter*-observer agreement on the Tabár classification. However, substantial to almost perfect *intra*-observer agreement has been reported previously [27,28]. In spite of the more intuitive approach, we found the overall inter-observer consistency to be highly comparable with the use of the BI-RADS scale. On the contrary, no obvious systematic disagreement was demonstrated. Consistency was highest for Pattern II which can be explained by the fact that fatty breasts are easier to assess and PII is a more frequent pattern. Still, a systematically PI/PII disagreement was seen which can be due to different perceptions of the amount of fibroglandular tissue (<20% dense tissue for Pattern II). Discrepancy was most evident for the borderline PI/PIV patterns, and 10% of the women would have been allocated differently (on a high/low-risk scale) by the two readers primarily because of this. Inconsistency between readers can, besides inherent variance, be explained by inconsistency in definition of the classification (when are nodular densities enlarged and how many are required to be classified as Pattern IV, when are structures judged visible in a very dense breast, perception of percentage density limits etc). Again, this is largely a matter of perception of the mammographic structures which is also influenced by the reader’s experience as a breast radiologist.

Zulfiqar et al divided the broad Pattern I into three sub-patterns based on density in a study exploring density among Malaysian women [43]. Subdivision of patterns or more extensive definitions could improve preciseness, on the other hand, the classification would be more difficult to adopt and it is doubtful if reproducibility would increase.

### PMD

Reliability between readers is reported to be stronger for computer-assisted interactive techniques than by visual assessment of density [4,44]. Boyd et al demonstrated an agreement between readers of ICC = 0.94 measured by the Cumulus software on CC views [2] and, likewise, Stone et al showed an ICC of 0.91 on MLO views [45]. We found a similar inter-observer agreement of ICC = 0.93 based on an average of both views. Despite the high inter-observer correlation the computer-assisted method still has a considerable subjective component. This is best illustrated graphically (Figure 4) where a *non-*systematic variance ranging from −11.1% till +12.9% is seen. The discrepancy (most differences within the range of one PMD quartile) is probably mainly explained by the two readers’ judgment of what represents dense area, but outlining the breast may also contribute to the variance (see also the limitations section). On a high/low-risk basis only 5% of the women would have been allocated differently by the two readers.

Generally, concordance tended to be lower for the right breast mammograms for all three density methods. We do not have a plausible explanation for this as left and right mammograms from each woman were acquired and processed in the same way by the same radiographer.

### Relative risk of breast cancer

Our study supports prior evidence that density patterns are associated with breast cancer risk [4]. On a multiple-category scale the three methods seemed to be influenced differently by the otherwise comparable level of inter-observer agreement. Especially, the categorical Tabár scale showed quite varying odds ratios for the two readers. On the other hand, disagreement regarding the BI-RADS classification didn’t show any impact on OR estimates, which were consistent among the readers and comparable with ORs found by others [4,13]. McCormack et al reported combined relative risks (RRs) of 2.04 for BI-RADS D2, 2.81 for D3 and 4.08 for D4 from two studies [4]. According to the quantitative PMD measure the same authors reported pooled RRs of 1.79, 2.11, 2.92 and 4.64 for percentage density 5-24%, 25-49%, 50-74% and 75-100% (compared with the reference category of <5%), respectively [4]. These RRs are also comparable with our results, but it should be noted that the *cut offs* between categories and the reference categories are not the same between studies (we use quartiles based on equal percentage ranges with a reference category of < ≈18%).

An interesting finding is that pattern IV by Tabár demonstrated the highest ORs (including the highest number of cases categorized to the high-risk group) of all the patterns investigated, even in spite of the inter-observer variance. The specific association with PIV was also found by Jakes and colleagues in an Asian population [39]. They demonstrated an unadjusted OR of 2.59 when PIV was compared with the combined group of Tabar’s pattern I, II, III and V, which was also seen consistently (and significantly) after adjusting individually for other breast cancer risk variables and confounders. They found the pattern to be associated with nulliparity, high educational status and grade 3 cancers. For comparison we found ORs of 2.85 by Reader1 and 3.15 by Reader2 when the low-risk category was changed to include Pattern V as well.

We saw that divergence in relative risk estimates between readers diminished almost completely after categorising into only two risk-groups. Grove et al investigated the effect of “misclassification” of Wolfe’s mammographic classification and argued that the overall concordance is not as important as the specific type of misclassification in estimating risk. Moreover, they stated that risk ratios are very sensitive to misclassification and risk ratios of 2 or 3 can be expected on a high/low-risk categorization even though “true” risk ratios may be quite high, which is in agreement with our findings [46]. We also found that even though the proportion of cases in the high-risk groups was similar for both readers, the actual number of women categorized to each risk-group did differ, which was most pronounced for the BI-RADS scale. Likewise, the number of women categorized with a high-risk density pattern differed between methods of assessment. It is important to be aware of this in a potential personalized screening set up. In total only 23% of the women would consistently have been classified in the high-risk group by all three methods by R1 and 22% by R2. It is beyond the scope of this article to draw conclusions on, if this can partly be explained by the fact that the three methods may catch different risk parameters.

### Strengths and limitations

We consider the use of two-view screening mammograms a strength of our study. As argued by others density of the breast should be perceived as a volume rather than an area [23-25,37], which has been well illustrated by Ciatto and co-workers [25]. On the other hand, studies have shown that there appears to be no difference in using an average of two or more mammograms compared with either of the two single views (CC or MLO) using the computer-assisted technique [47,48]. A study on visual assessment of PMD, found that the magnitude of breast cancer risk association was significantly increased using both views compared with only MLO alone, though [37]. Our CC and MLO views correlated well and we decided to use an average of both views in this study. Our multifaceted statistical evaluation of the quantitatively measured PMD, using the Pearson correlation coefficient, ICC, Bland-Altman and scatter plot, is also considered a strength. The frequent use of the Pearson correlation coefficient alone only provides a one-dimensional picture of the degree of agreement as discussed in detail by Abdolell et al [49]. Moreover, we find it a strength of this study to have included the qualitative Tabár classification and demonstrated its reproducibility. With ACR’s new definition on the BI-RADS density classification (5^th^ edition) returning from a more quantitative to a qualitative classification, it seems as if the more qualitative classifications also have a role to play in the future.

We recognise our study also has some limitations to be addressed: In this retrospective study on a screening cohort we have not been able to control for other breast cancer risk variables other than age. However, from a clinical point of view the question is what we can do with the information available to us, if we were to do risk-based stratification of screening women. In many screening programmes—like ours—the only information available to us is the woman’s age and her mammogram. Therefore, ORs have not been adjusted for other risk factors such as BMI, history of breast cancer, menopausal status, and other reproductive variables in this study. The ORs should obviously be interpreted with precaution when compared with other studies, and are in the present study primarily to be compared between readers. BMI is known to be one of the most important confounders; however, the lack of adjustment for BMI has probably led to some underestimation of risk [4,50]. Moreover, we did not differentiate between interval cancers (defined as cancers diagnosed between two screenings) and screen-detected cancers. We might have included some “excess” cancers which may have been initially un-detected (masked at the negative screening in 2007), leading to an overestimation of risk [4].

In addition, readings were done on analogue digitized mammograms reducing the quality of the images. Mammograms were rather dark and, accordingly, the breast boundary was not easy to delimit and might have influenced PMD estimation. The readers also had to compensate for colouring artefacts (e.g. from the pectoral muscle) when setting the threshold. Accuracy and reliability of methods for density assessment on *digital* mammograms (including automatic techniques) may be superior. However, important information from film-based mammograms still exists. We believe this will be of interest for epidemiological long term follow-up studies for many years to come.

Finally, it would have strengthened our study methodologically to have had more readers. Keeping the above limitations in mind we did find our results to be comparable to others, though.

## Conclusions

Our study shows that the qualitative Tabár classification has comparable inter-observer reproducibility with well tested density methods, and confirms the association between Tabár’s PIV and breast cancer.

Regardless of substantial to almost perfect inter-observer reproducibility for all three methods investigated, different impact on relative risk estimation in terms of ORs for breast cancer is seen on a multiple-category scale. Even though, risk estimates become more uniform on a high/low-risk scale, the consistency of women with a high risk pattern differs between both readers and methods.

A more detailed definition on classification criteria, an expanded set of reference images or a proficiency test may improve inter-observer agreement to some degree using these manual methods. However, it is doubtful if it is possible to ensure and maintain this high standardization within different breast imaging units and in the screening setting.

Thus, an automated, objective and reproducible method to estimate density or texture (or both) from the mammogram are needed to fully overcome the impact of subjectivity. Our study is based on analogue images. However, many breast imaging units have in recent years switched to digital mammography. This has encouraged the development and improvement of fully automated techniques, which has been shown to be valid alternatives on digital mammography [32,33]. In addition, the applicability of other imaging modalities for density assessment is being investigated including DBT and MRI [34,35,51]. The numerous methodologies existing today may capture different aspects of density, and it remains unresolved which particular methods to use. This will necessarily depend on the aim (research/clinic/tailored screening). However, it is evident that different methods are not interchangeable.

In conclusion, our study confirms that improvement of fully automated methods should be continued to overcome subjectivity (as well as time consumption) in measuring density for research and clinical risk assessment.

## Abbreviations

ACR: The American College of Radiology
BI-RADS: Breast imaging reporting and data system
CC: Craniocaudal
CI: Confidence interval
DBT: Digital breast tomosynthesis
DCIS: Ductal carcinoma in situ
ICC: Intraclass correlation coefficient
LAL: Lower agreement limit
MLO: Mediolateral oblique
NS: Non-significant
OR: Odds ratio
PMD: Percentage mammographic density
R1: Reader 1
R2: Reader 2
UAL: Upper agreement limit
